# Regular Chinese Green Tea Consumption Is Protective for Diabetic Retinopathy: A Clinic-Based Case-Control Study

**DOI:** 10.1155/2015/231570

**Published:** 2015-10-11

**Authors:** Qinghua Ma, Dandan Chen, Hong-Peng Sun, Ning Yan, Yong Xu, Chen-Wei Pan

**Affiliations:** ^1^Jiangsu Key Laboratory of Preventive and Translational Medicine for Geriatric Diseases, School of Public Health, Medical College of Soochow University, Suzhou 215123, China; ^2^The 3rd People's Hospital of Xiangcheng District, Suzhou 215134, China; ^3^School of Radiation Medicine and Protection, Medical College of Soochow University, Suzhou 215123, China; ^4^Collaborative Innovation Center of Radiation Medicine of Jiangsu Higher Education Institutions, Suzhou 215123, China

## Abstract

*Objective.* To determine the association between regular Chinese green tea consumption and the risk of diabetic retinopathy (DR) in diabetic patients in China. *Methods.* 100 DR patients and 100 age-sex-matched diabetic controls without retinopathy were recruited in a clinic-based, case-control study. DR was defined from retinal photographs and detailed information on Chinese green tea consumption of the participants was collected through a face-to-face interview. *Results.* The crude odds ratio [OR] of Chinese green tea consumption for DR was 0.49 (95% confidence interval: 0.26–0.90). When stratified by sex, the protective effect of Chinese green tea consumption on DR was statistically significant in women (*P* = 0.01) but not in men (*P* = 0.63). After adjusting for age, sex, and other confounders, DR was significantly associated with Chinese green tea consumption (OR = 0.48; *P* = 0.04), higher systolic blood pressure (OR = 1.02; *P* = 0.05), longer duration of diabetes (OR = 1.07; *P* = 0.02), and the presence of family history of diabetes (OR = 2.35; *P* = 0.04). *Conclusions.* Diabetic patients who had regularly drunk Chinese green tea every week for at least one year in their lives had a DR risk reduction of about 50% compared with those who had not. Regular Chinese green tea consumption may be a novel approach for the prevention of DR.

## 1. Introduction

Diabetic retinopathy (DR) is a common microvascular complication of diabetes and one of the major causes for visual impairment [1–5]. Globally, DR accounts for about 5% of all blindness, affecting 2 million people, and is the leading cause for blindness in people aged 15 to 64 years in industrialized countries [6]. Clinical treatment for DR such as ocular antivascular endothelial growth factor therapy in low-income countries is still a great challenge as the economic cost is considerable. Understanding the risk or protective factors for DR, especially modifiable ones, is crucial to enable the eventual prevention of this eye condition. Epidemiological studies have provided evidence that some modifiable factors including poor glycemic control and hypertension might play an important role in the development and progression of retinopathy in diabetic individuals [7].

Green tea is a popular beverage in East Asian communities such as China, Japan, Vietnam, and Thailand and has been reported to delay or prevent many clinical disorders such as cancers [8–11], osteoarthritis [12], and cardiovascular diseases [13, 14]. Green tea has also been suggested to decrease the risk of type 2 diabetes mellitus and is associated with decreased insulin resistance [15]. Green tea contains huge amount of polyphenols, which display strong antioxidant activity and anti-inflammatory effects [16]. Both oxidative stress [17, 18] and inflammation [19] have been strongly implicated in the pathogenesis of DR. Epigallocatechin-3-gallate, the most abundant and potent green tea catechin, is classified as an antioxidant based upon its chemical structure and has been extensively studied for its beneficial effects for health. In diabetic rats, epigallocatechin-3-gallate could not only reduce the level of anion production but also prevent the formation of acellular capillaries and pericyte ghosts, which may be beneficial to the retina [20]. Recently, some other compounds have been reported to show preventive effects on retinopathy. For example, *α*-mangostin has been reported to significantly and dose-dependently reduce the formation of retinal oxidative stress in hypoxia-treated retinal endothelial cells [21]. In addition, cocoa enriched with polyphenol could also improve the retinal sirtuin pathway, thereby protecting the retina from diabetic milieu insult [22]. Therefore, one can hypothesize that regular green tea consumption may be a modifiable protective factor for DR in humans. To our best knowledge, the effect of regular green tea consumption on the risk of DR has not been investigated in epidemiologic studies previously.

In this effort, we conducted a clinic-based case-control study to examine the possible protective effect of regular Chinese green tea consumption on the risk of DR in Chinese diabetic patients in Suzhou in eastern China, where the provenance of Chinese green tea,“Bi Luo Chun,” is located.

## 2. Materials and Methods

### 2.1. Study Population

The study population consisted of 100 DR patients and 100 age-sex-matched diabetic controls without DR. All cases and controls were selected through a medical record review of the diabetes clinic in the 3rd People's Hospital of Xiang Cheng District, Suzhou, China in the year 2013. As the cases and controls were age-sex-matched, it was difficult to find more matched pairs through the medical record review. We included study subjects if they were 18 years or older and had a previous physician diagnosis of type 2 diabetes mellitus. Exclusion criteria included those who did not give consent to take part in this medical review, those with previous ocular trauma or surgery, and those with other clinically significant ocular comorbidities. Written informed consent was obtained from the study subjects and ethics approval was obtained from the Institutional Review Board of the Medical College of Soochow University. The study was conducted in accordance with the tenets of the World Medical Association's Declaration of Helsinki.

### 2.2. Measurement of Diabetic Retinopathy

All subjects in the case and control group undertook retinal fundus examination performed by experienced ophthalmologists at the time they visited the clinic. The examination included slit-lamp biomicroscopy (Zeiss SL 115 Classic Slit Lamp, Carl Zeiss Meditec AG Jena Germany) and dilated retinal examination using 60D 174 aspheric condensing lens (Volk) and binocular indirect ophthalmoscopy (BIO; Keeler all pupil) with a 20D lens. Retinal fundus photographs were taken using a fundus camera (Type CR6-45NM, Canon Inc., USA) and were used for DR grading by an experienced ophthalmologist. Macular edema was defined by hard exudates in the presence of microaneurysms and blot hemorrhage with one disc diameter from the foveal center or presence of focal photocoagulation scars in the macular areas. Diabetic retinopathy was defined as the presence of microaneurysms, dot-blot hemorrhages, intraretinal microvascular anomalies, new vessels on the disc or elsewhere, cotton-wool spots, exudates, and clinically significant macular edema based on the Early Treatment Diabetic Retinopathy Study (ETDRS) classification [23]. The detailed grading schemes for DR are described in Table 1. If retinal images were not available or readable due to media opacity, findings recorded by the examining ophthalmologists were used.

### 2.3. Measurement of Chinese Green Tea Consumption

After the medical review, the selected study participants were invited to the clinic or were interviewed at home based on their preferences. All selected study participants participated in the interview. Detailed information on regular Chinese green tea consumption of the participants was collected by a research assistant through a face-to-face interview. First, we asked “Have you ever had Chinese green tea every week for at least one year in your life?” If the answer was “yes,” the participant was defined as a “regular Chinese green tea drinker.” Otherwise, he or she was defined as a “nonregular Chinese green tea drinker.” For regular Chinese green tea drinkers, we further asked several questions including “How many years have you drunk Chinese green tea every week?”; “How many glasses of Chinese green tea do you usually drink per day in the past 12 months?”; “When did you start drinking Chinese green tea every week?”; and “Are you still drinking Chinese green tea every week now?”.

### 2.4. Measurement of Confounders

All participants also underwent a detailed interview and information on education, lifestyle risk factors (e.g., smoking, alcohol intake and physical activity), family history of diabetes, and medication use was collected by a trained interviewer. Before the interview, the purpose of the study was explained and the participants were assured that the information provided would be strictly confidential. To control the quality of data and minimize the potential information bias, the interviewer was not informed if the participant was in the case or control group. Height was measured in centimeters using a wall-mounted measuring tape after removing shoes and weight was measured in kilograms using a digital scale after removing heavy clothing. Body mass index (BMI) was defined as weight divided by the square of height in meters (kg/m^2^). Systolic and diastolic blood pressures were measured using a digital automatic blood pressure monitor (Dinamap model Pro100V2, Norderstedt, Germany). Clinical data including duration of diabetes, fasting blood glucose when diabetes was diagnosed, and if the participant had undergone insulin therapy were retrieved from medical record review.

### 2.5. Statistical Analyses

Statistical analysis was performed using SPSS version 17.0 (SPSS, Inc., Chicago, IL). The demographics and risk factors between cases and controls were compared using independent *t*-tests or chi-square tests according to the category of the data. A 2 by 2 table was constructed to obtain the crude odds ratio (OR) of regular Chinese green tea consumption for DR in the overall study participants and then it was stratified by sex. Among regular Chinese green tea drinkers, the duration of Chinese green tea consumption and number of glasses of Chinese green tea consumption per day were compared between the cases and controls using independent *t*-tests. Multiple logistic regression analyses were performed to determine the association of regular Chinese green tea consumption (independent variables) with DR (dependent variables), adjusting for potential confounders.

## 3. Results

In total, 200 diabetic patients including 68 men and 132 women aged 35 to 85 years were included in the analysis (mean age: 64.8 ± 9.0 years for both cases and controls). Among the 100 cases with DR, 94 had minimal or mild nonproliferative diabetic retinopathy (NPDR), 3 had moderate NPDR, and the other 3 had sight-threatening retinopathy (STDR). The fasting blood glucose level was significantly lower in participants with a regular Chinese green tea drinking history compared with those without (7.40 versus 7.99 mmol/L; *P* < 0.001).  Table 2 shows the characteristics of 100 cases and 100 age-sex-matched controls. Compared with controls, cases had a higher systolic blood pressure (*P* = 0.04) and a longer duration of diabetes (*P* = 0.04) and were more likely to have family history of diabetes (*P* = 0.02). There were no significant differences in education, height, weight, BMI, diastolic blood pressure, smoking status, alcohol intake, insulin therapy, physical activity, and fasting blood glucose (all *P* > 0.05).

Table 3 shows the distribution of regular Chinese green tea drinkers in the case and control groups, respectively. In the overall study participants, 23 of 100 DR patients had a regular Chinese green tea drinking history, compared with 38 of 100 in the controls. The crude OR of regular Chinese green tea consumption for DR was 0.49 (95% confidence interval (CI): 0.26–0.90). When stratified by sex, the protective effect of regular Chinese green tea consumption on DR was statistically significant in women (OR = 0.32; 95% CI: 0.13–0.75; *P* = 0.01) but not in men (OR = 0.79; 95% CI: 0.30–2.06; *P* = 0.63).

**Figure 1:**
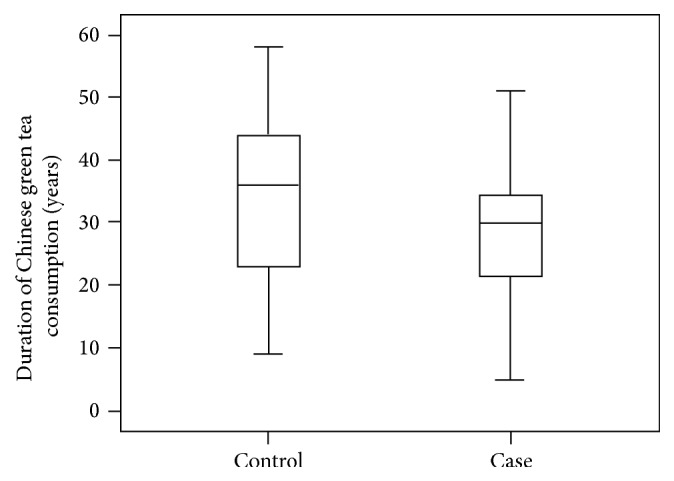
Comparison of mean duration regular Chinese tea consumption between the case and control group.

**Figure 2:**
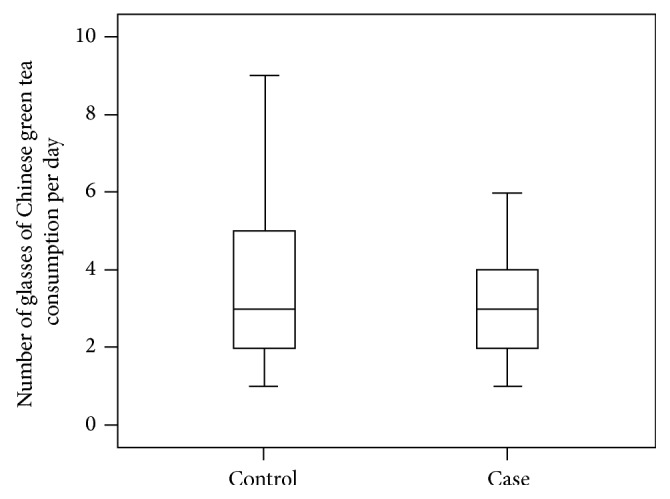
Comparison of mean amount of regular Chinese tea consumption per day between the case and control group.

Among the participants who had regularly drunk Chinese green tea every week for at least one year, subjects without DR had a longer duration of Chinese green tea consumption (33.8 versus 28.9 years; Figure 1) and more glasses or drinks per day (3.77 versus 3.17 glasses; Figure 2). However, these differences between the two groups were not statistically significant (*P* > 0.05).

Associations of DR with regular Chinese green tea consumption and other risk factors were further examined in a multiple logistic regression model and the results are shown in Table 4. In multivariate analysis, DR was significantly associated with regular Chinese green tea consumption (OR = 0.48; 95% CI: 0.24–0.97; *P* = 0.04), higher systolic blood pressure (OR = 1.02; 95% CI: 1.00–1.05; *P* = 0.05), longer duration of diabetes (OR = 1.07; 95% CI: 1.01–1.14; *P* = 0.02), and the presence of family history of diabetes (OR = 2.35; 95% CI: 1.03–5.35; *P* = 0.04). No joint effect of regular Chinese green tea consumption with other risk factors on the risk of DR was found (all *P* > 0.05).

## 4. Discussion

This paper reported on the relationship of regular Chinese green tea consumption with DR in a clinic-based case-control study in eastern China. Diabetic patients who had regularly drunk Chinese green tea every week for at least one year in their lives had a DR risk reduction of about 50% compared with those who had not. The study suggests a possible protective effect of regular Chinese green tea consumption on the risk of DR and may have important public health implications for DR prevention. This observation needs to be confirmed in well-designed cohort studies.

The conclusion that green tea, when consumed on a regular basis, is beneficial to health and could reduce the risks of major metabolic syndrome including obesity, diabetes, and cardiovascular diseases seems to have been well established. For example, in a cohort study in Japan, a one-third risk reduction of developing type 2 diabetes mellitus was found in subjects consuming 6 or more cups of green tea on a daily basis compared with those consuming only less than 1 cup per week [24]. Another population-based prospective cohort study in Japan found that green tea consumption is associated with reduced mortality due to cardiovascular disease [25]. An inverse relationship among habitual green tea consumption, percent body fat, and body fat distribution was found in Taiwanese adults, especially for those who have maintained the habit of tea consumption for more than 10 years [26]. However, epidemiologic evidences in humans supporting a protective effect of regular Chinese green tea consumption on DR are limited and our study is the first one to report this protective effect. Our study subjects were all patients with type 2 diabetes mellitus in the same clinic and the cases and controls were strictly matched by age and sex, which aimed at minimizing the effect of potential confounders. The risk of DR was reduced by about 50% in subjects with a regularly Chinese green tea consumption history compared with those without. Although we have to acknowledge that the magnitude of risk reduction in a case control design is always exaggerated and the effect size would be smaller in a population-based cohort study on the same research topic, we believe that even smaller effects on an individual basis could have a large public health impact considering the popularity and high rate of green tea consumption in China and other East Asian communities. Therefore, our study may suggest a novel approach in prevention of DR in East Asia.

Animal experiments may shed some light into the possible mechanism behind the observed association in this study. In an animal experiment [27], the expression of glial fibrillary acidic protein, oxidative retinal markers, and glutamine synthetase levels were all found to be increased in diabetic rats while occludin and glutamate transporter and receptor were decreased. Müller cells exposed to high-glucose medium produced higher levels of reactive oxygen species and glutamine synthetase but reduced levels of glutathione, glutamate transporter, and glutamate receptor. ARPE-19 cells exhibited increased reactive oxygen species production accompanied by decreased expression of claudin-1 and glutamate transporter. Treatment with green tea fully restored all the above-mentioned alterations in diabetic rats and in retinal cells. The authors concluded that green tea protected the retina against glutamate toxicity via an antioxidant mechanism. In another study [28] which aimed to investigate the effect of green tea on diabetes-induced retinal oxidative stress and proinflammatory parameters in rats, antioxidant enzymes showed a more than 2-fold decrease in activity in diabetic retina compared with normal retina. Both superoxide dismutase and catalase enzymatic activities were restored close to normal in the treated group by green tea. Expression of proinflammatory parameters including tumor necrosis factor-*α* and vascular endothelial growth factor was significantly inhibited in green tea-treated retina as compared to diabetic retina. These findings of animal studies suggested the possible mechanism of a beneficial effect of green tea in the prevention and treatment of DR.

Our study may have significant implications for DR prevention. Many diabetic patients with retinopathy, especially in low-income countries, do not have adequate access to clinic treatment. For example, patients with severe DR in rural China were estimated to be around 2.4 million and most of them have not been properly treated, largely because of a lack of medical resources and the ability to afford the high economic cost for treatment [29]. To address this public health issue, a low-cost, safe, and easy-to-operate approach for DR prevention is needed. Chinese green tea is a cheap and easy-to-access beverage in China and other nations in East Asia. If the protective effect of regular Chinese green tea consumption on retinopathy in diabetic individuals is confirmed, there may be a pressing need for implementing health promotion programs on the potential benefit of green tea consumption among high DR risk population groups in these areas.

The main strength of our study includes a standardized DR grading approach and the proper design with an age-sex-matched sample in the same diabetes clinic to minimize the selection bias in classical case control studies from an epidemiologic perspective. Limitations of this study should also be noted. First, as a clinic-based study, it should be born in mind that the study participants may be unrepresentative of the general population. The potential biases in clinic-based studies could distort the findings. Second, information on Chinese tea consumption was collected using a predesigned questionnaire. Although we had tried our best to control the quality of data, information bias during the interview could not be excluded. Third, we did not collect the data of diet in this study. The frequent consumption of Chinese green tea may be simply a marker for a lifestyle, possibly a more traditional Chinese lifestyle including a more traditional Chinese diet, which includes less consumption of “junk food” or high sugar foods. Finally, the sample size was small which makes the results less cogent. We found the association was significant in women but not in men, but we did not believe that sex was the effect modifier for the observed association between regular Chinese green tea consumption and DR. This is probably due to the small sample size in men, resulting in a lack of statistical power for detecting a significant finding. Longitudinal data with sufficient sample size and proper study design are warranted to confirm the relationships of baseline regular green tea consumption with incident DR.

## 5. Conclusions

In summary, we reported that Chinese diabetic individuals who had a regular Chinese green tea drinking history were at a lower risk of DR compared with those who did not, suggesting that Chinese green tea consumption may be a cheap and easy-to-operate approach for DR prevention in low-income countries. Longitudinal cohort studies are warranted to confirm this finding and randomized control trials are warranted to examine the effectiveness and cost-effectiveness of regular Chinese green tea consumption in diabetic populations to decrease the risk of DR in general populations.

## Figures and Tables

**Table 1:** Grading schemes of diabetic retinopathy.

Severity of diabetic retinopathy	Grading schemes
Minimal nonproliferative diabetic retinopathy (NPDR)	Level 15 through 20
Mild NPDR	Level 35
Moderate NPDR	Level 43 through 47
Severe NPDR	Level 53
Proliferative diabetic retinopathy (PDR)	Level more than 60
Macular edema	Hard exudates in the presence of microaneurysms and blot hemorrhage with one disc diameter from the foveal center or presence of focal photocoagulation scars in the macular areas.
Clinically significant macular edema (CSME)	Macular edema within 550 *µ*m of the foveal center or if focal photocoagulation scars were present in the macular area.
Sight-threatening retinopathy (STDR)	Severe NPDR, PDR, or CSME.

**Table 2:** Characteristics of study population.

	Cases (*n* = 100)	Controls (*n* = 100)	*P* value
Education			
No formal education	28 (28)	33 (33)	0.68
Primary education	39 (39)	31 (31)	
Secondary education	21 (21)	24 (24)	
Tertiary education	12 (12)	12 (12)	
Height (cm)	161.3 (7.8)	161 (7.3)	0.82
Weight (Kg)	62.4 (9.9)	62.2 (9.7)	0.90
Body mass index	23.9 (3.1)	24 (3.8)	0.81
Systolic blood pressure (mmHg)	134.9 (14.7)	130.9 (11.7)	**0.04**
Diastolic blood pressure (mmHg)	83.4 (8.8)	82.9 (8.1)	0.72
Smoking status			
No smoking history	75 (75)	70 (70)	0.61
Past smoker	12 (12)	12 (12)	
Current smoker	13 (13)	18 (18)	
Alcohol drinking			
No alcohol drinking history	86 (86)	86 (86)	0.18
Past alcohol drinker	5 (5)	1 (1)	
Current alcohol drinker	9 (9)	13 (13)	
Duration of diabetes (years)	8.9 (5.3)	7.3 (5.4)	**0.04**
Insulin therapy	15 (15)	19 (19)	0.45
Family history of diabetes	24 (24)	11 (11)	**0.02**
Physical activity	34 (34)	39 (39)	0.46
Fasting blood glucose (mmol/L)	7.43	7.73	0.34

Bold type indicates statistical significance (*P* < 0.05).

Data presented are means (standard deviations) or number (%), as appropriate for variable.

**Table 3:** Crude odds ratio of regular Chinese green tea consumption for diabetic retinopathy in the overall study participants and stratified by sex.

Number of subjects	All			Male			Female		
	Case	Control	Total	Case	Control	Total	Case	Control	Total
Regular Chinese green tea (+)	23	38	139	14	16	30	9	22	31
Regular Chinese green tea (−)	77	62	61	20	18	38	57	44	101
Total	**100**	**100**	**200**	**34**	**34**	**68**	**66**	**66**	**132**

Odds ratio	0.49			0.79			0.32		

95 CI	0.26, 0.90			0.30, 2.06			0.13, 0.75		

*P* value	0.02			0.63			0.01		

**Table 4:** Multivariate analysis on the associations of regular Chinese green tea consumption and other potential risk factors with diabetic retinopathy.

		Odds ratio	95% Confidence interval	*P*
Regular Chinese green tea consumption	Ever versus never	0.48	0.24, 0.97	0.04
Education	Formal versus no formal education	1.26	0.65, 2.43	0.50
Body mass index	Per unit increase	1.02	0.93, 1.12	0.62
Systolic blood pressure	Per mmHg increase	1.02	1.00, 1.05	0.05
Smoking history	Ever versus never	0.83	0.37, 1.85	0.64
Alcohol drinking	Ever versus never	1.29	0.52, 3.21	0.59
Duration of diabetes	Per year increase	1.07	1.01, 1.14	0.02
Insulin therapy	Ever versus never	0.73	0.33, 1.62	0.44
Family history of diabetes	Present versus absent	2.35	1.03, 5.35	0.04
Physical activity	Often versus rare	0.85	0.45, 1.62	0.64
Fasting blood glucose	Per mmol/L increase	0.93	0.80, 1.07	0.29
